# Case Report: Spontaneous cholecystocutaneous fistula, a rare cholethiasis complication

**DOI:** 10.12688/f1000research.12235.1

**Published:** 2017-09-27

**Authors:** Nunzio Maria Angelo Rinzivillo, Riccardo Danna, Vito Leanza, Melissa Lodato, Salvatore Marchese, Francesco Basile, Guido Nicola Zanghì

**Affiliations:** 1 Department of Surgery, Policlinico Vittorio Emanuele University Hospital, University of Catania, Catania, Sicily, 95100, Italy

**Keywords:** Cholecystocutaneous, Fistula, cholethiasis, abdominal fistula, gallbladder stones, gallbladder cancer, Bile Ducts, Laparotomy

## Abstract

One of the most unusual complications in cholethiasis is spontaneous cholecystocutaneous fistula, which has only been reported a few times in the literature.  We report the case of a 76 year old man who presented with a right hypochondrium subcutaneous abscess, with pain evoked through palpation.  No comorbidity in the patient’s medical history were noted.   Confirmation of cholecystocutaneous fistula was made using the proper diagnostic process, which is computed tomography with contrast media, followed by hepatobiliary MRI. This confirmed the presence of a fistulous pathway between the gallbladder and the skin.  The patient underwent cholecystectomy surgery and open laparotomy with *en block* aponeurotic muscle, skin and fistula orifice excision.

## Introduction

Spontaneous cholecystocutaneous abscess or fistula is an extremely uncommon complication of gallbladder disease. Less than 100 cases have been described in the literature. The first descriptions of cholecystocutaneous fistula was made by Thelisus in 1670. Later Courvoisier reported 169 cases of biliary fistula in the 19th century ^1^. The natural history of the disease has changed from suppurative cholecystitis with spontaneous rupture to operative external drainage of an abscess ^2^. Early and effective medical and surgical management of biliary tract disease can prevent this rare condition. Stone obstruction of the biliary tree plays a crucial role in the pathophysiology of the development of this condition; intra-gallbladder pressure can increase dramatically due to the obstruction of the cystic duct. The unresolved obstruction of the bile outflow compromise gallbladder wall blood circulation, as well as lymphatic drainage, resulting in necrosis of the gallbladder wall with the fistula formation. Once pierced, the gallbladder may drain into the peritoneal cavity, causing peritoneal localized abscess or the abscess can lead into an external fistula due to its adherence to the abdominal wall ^1– 3^.

## Case report

**Figure 1.**
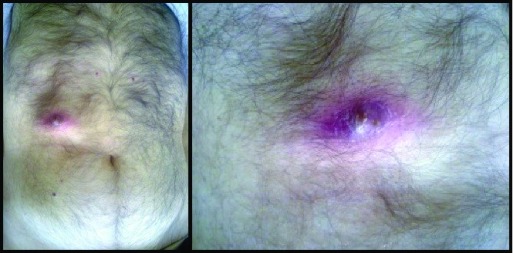
Cholecystocutaneous fistula, macroscopic appearance.

A 76 year old man was admitted to our University Hospital “Ospedale Vittorio Emenuele” and seen in our surgical department. He presented with a 3 cm tumefaction of the right hypochondrium, surrounded by an erythematous skin area, with small secretion of a yellowing-green material, attributable to a bile leaking ( Figure 1). The patient’s medical history was clear from previous medical disease and surgery; he only referred to previous upper right quadrant pain and nonspecific dyspeptic disorder.

An abdominal ultrasound examination revealed the presence of a lesion in the aponeurotic muscle wall, but the possible underlying pathology was unknown. No other signs of pathology were observed. Routine blood work was normal.

**Figure 2.**
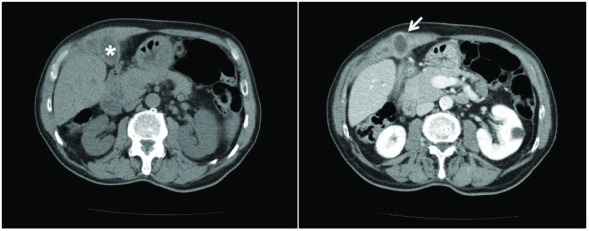
Abdominal computed tomography images. Left image, * indicates the gallbladder; right image, the arrows indicates the fistula.

**Figure 3.**
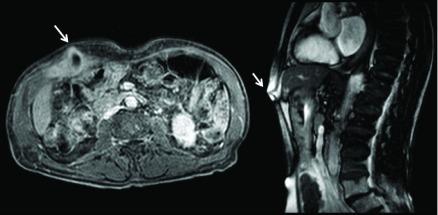
Hepatobiliary MRI. The white arrows show the fistula. From left to right: coronal and sagittal fistula view.

Abdominal computed tomography scan with contrast media showed the gallbladder walls had diffuse thickened and blurred edges, and the right and transverse abdominal muscles were almost covered and embedded with minute hypo-dense ailments compatible with relapsing phlogistic processes ( Figure 2). Hepatobiliary MRI detected that the gallbladder had anteriorly shifted and adhered to the right abdominal muscles. The side wall showed a break through with consensual purulent collection, which extruded through the thick abdominal wall ( Figure 3). Eventually, several different-sized stones were revealed inside the cholecyst. Consequently, a diagnosis of spontaneous cholecystocutaneous fistula was made.

The patient underwent cholecystectomy surgery 10 days after diagnosis, with an open laparotomy with *en block* aponeurotic muscle, skin and fistula orifice excision. In order to have a good abdominal wall reconstruction, a properly shaped Prolene Prosthesis was placed using fibrin glue ^4– 7^. The patient received broad-spectrum antibiotics after surgery (Piperacillin/Tazobactam 4.5 g, 3 times a day for 5 days via IV).

 Considering the patient’s good general condition and good post-operative course, discharge was on the seventh day post-surgery. Surgical wound re-dressing was made one week after discharge in our facility, where the surgical stitches were removed. Scar appearance was good and without concern. No other dressing was needed and the scar was covered with a bandage. The first follow-up was scheduled 15 days after discharge, second one 60 days from discharge. For both follow-ups, routine blood work and surgical scar checking were performed, the results of which were normal and the scar was healing normally. 

A histological examination confirmed the diagnosis of chronic cholecystitis with gallstones and cholecystocutaneous fistula.

## Discussion

Thanks to the progress made with medical imaging and surgical techniques, biliary fistula is today a very rare pathology ^8– 11^. Fistulas often represent the result of post-surgical ^12^ or post-traumatic ^15^ complications that generally involve the duodenum (77%) and colon (15%) ^16^.

Spontaneous cholecystocutaneous fistula represents a truly exceptional event, as confirmed by the analysis of the literature, which revealed only 28 cases published over the last 10 years ( Table 1). This disease mainly affects female subjects over the age of 60. Etiology is generally due to an acute inflammatory process as a consequence of a cholecystitis or chronic gallstones disease ^17– 20^, although there are described cases of spontaneous cholecystocutaneous fistula in the absence of gallstones ^21^. Rarely does cholecystocutaneous fistula evolve into a neoplastic process. Instead, fistula can be a sign of gallbladder cancer ^19, 20^. According to Sibakoti, polyarteritis nodosa with gallbladder vasculitis and prolonged use of high dose steroids can be considered predisposing factors ^21^. Fistula *primum movens* is by cystic duct obstruction, which increases the pressure within the gallbladder, with wall distension and impaired vascularization, resulting in the formation of focal necrosis of the wall with perforation evolution and abscess formation to the surrounding area that will rupture in to the continuous structures. In the present case, the abscess drained through the abdominal wall and the fistulose pathway originated from the bottom of the gallbladder. This area is the most distant from the cystic artery and physiologically the least vascularized and therefore more susceptible to ischemia ^17^.

**Table 1.** Publications within the last 10 years concerning cholecystocutaneous fistula.

Author(s)	Year published	Number of cases	Country	Age	Gender	Treatment technique
Maynard *et al.* ^35^	2016	1	United Kingdom	68	F	Open
Jayasinghe *et al.* ^36^	2016	1	United Kingdom	>70	F	Open
Guardado-B *et al.* ^37^	2015	1	Mexico	30	F	Open
Álvarez *et al.* ^38^	2014	1	Argentina	79	F	Open
Dixon *et al.* ^39^	2014	1	United Kingdom	94	F	Open
Pripotnev and Petrakos ^40^	2014	1	Canada	85	F	Open
Kim *et al.* ^41^	2013	1	Australia			Open
Jayant *et al.* ^42^	2013	1	India	42	F	Open
Sodhi *et al.* ^43^	2012	1	India	66	F	Open
Kapoor *et al.* ^44^	2013	1	India	45	M	Open
Ozdemir *et al.* ^45^	2012	2	India	45 & 65	M	Open
Ugalde Serrano *et al.* ^46^	2012	1	Spain	83	M	Open
Andersen and Friis-Andersen ^24, 47^	2012	1	Denmark	89	F	Open
Ioamidis *et al.* ^48^	2012	1	Greece	71	M	Open
Cheng *et al.* ^49^	2011	1	China			Open
Gordon ^50^	2011	1	U.S.A.	83	F	Open
Sayed *et al.* ^51^	2010	1	United Kingdom	85	F	Open
Pezzilli *et al.* ^52^	2010	1	Italy	90	F	Open
Metsemakers *et al.* ^53^	2010	1	Belgium	69	M	Open
Tallón Aguilar *et al.* ^54^	2010	1	Spain	83	F	Open
Kahn *et al.* ^55^	2010	1	Ireland	76	M	Open
Hawari *et al.* ^56^	2010	1	United Kingdom	84	M	Open
Murphy *et al.* ^57^	2008	1	United Kingdom	80	M	Open
Ijaz *et al.* ^58^	2008	1	United Kingdom	80	F	Open
Chatterjee *et al.* ^59^	2007	1	India	45	F	Open
Malik *et al.* ^60^	2007	1	United Kingdom	76	F	Laparoscopic

The external fistular orifice is usually on the right upper quadrant, but other locations have been described, including the left hypocondrium, umbilical scar, right lumbar, and right iliac fossa, and rarely the right gluteus and breast region ^19, 24, 47^.

The diagnostic process always begins with upper abdomen ultrasound and ends with hepatobiliary MRI to visualize the biliary tree. Considering that 11% of cholecystitis have concomitant presence of gallstones in the main bile duct, it is advisable to perform endoscopic retrograde cholangiopancreatography (ERCP) ^2– 29^. In our case CT with CM and hepatobiliary MRI confirmed the fistula presence and it was not necessary to execute the ERCP before surgery. Although an intraoperative cholangiogram was performed to check that the bile ducts were clear from gallstones. Cholecystocutaneous fistula has always been treated by two different strategies. The first includes a two-step approach: percutaneous drainage and antibiotic therapy, and subsequently cholecystectomy. The second directly involves laparotomy cholecystectomy execution with *en block* aponeurotic muscles, as well as skin and fistula orifice excision.

 The second strategy is the most commonly used since the two-step approach treatment is reserved for patients with sepsis and poor general condition ^12, 15, 29^.

 In 1998, Kumar described the first case of gynecological fistula treated with laparoscopic technique, proposing to the scientific community the feasibility of this innovative approach ^27, 30^.

## Conclusion

Rarity of this pathology confirms the great quality of progress made by early diagnostic techniques and medical treatment to prevent complication of cholethiasis. Although cholecystocutaneous spontaneous fistula is not common, it can lead to a serious condition. If not quickly treated, it can rapidly evolve into a generalized septic state with severe impairment prognosis. In our case, the patient was in good health arguably because the fistula was draining the most of the abscess outside the body and not in the peritoneum space. Surgical treatment was, however, essential to restore the physiologic bile flow and adequate broad-spectrum antibiotic prophylaxis lowed the risk of post-operative infections. Although laparoscopic approaches have been described since 1998, this pathology is, in most cases, continuing to be treated with open technique, most likely because it is easier and with fewer risks of post-surgical complications ^31– 34^.

## Consent

Written informed consent was obtained from the patient for the publication of the patient’s clinical details and related images.
