# Supplementary material for: Case Report: Spontaneous cholecystocutaneous fistula, a rare cholethiasis complication
Source: F1000Res. 2017 Sep 27;6:1768. [Version 1] doi: 10.12688/f1000research.12235.1 (PMC5698921; doi:10.12688/f1000research.12235.1)
Supplement: Peer review history

# Peer review: Referee response for version 1

**DOI:** 10.5256/f1000research.13246.r28008

**Published:** 2017-11-24

**Authors:** Giuseppe Navarra

**Affiliations:** 1 Surgical Oncology Unit, Department of Human Pathology of Adult and Evolutive Age, University Hospital of Messina, Messina, Italy

The article Case Report: Spontaneous cholecystocutaneous fistula, a rare cholethiasis complication covers a subject that nowadays is quite uncommon. It goes through the background of the case’s history, it provides details of diagnostic tests, treatment given and outcomes reached. It could be useful for other practitioners. It can be accepted for indexing with minor English revision.

I have read this submission. I believe that I have an appropriate level of expertise to confirm that it is of an acceptable scientific standard.

# Peer review: Referee response for version 1

**DOI:** 10.5256/f1000research.13246.r28162

**Published:** 2017-11-21

**Authors:** Antonino Agrusa

**Affiliations:** 1 Department of General Surgery, Urgency and Organ Transplantation, University of Palermo, Palermo, Italy

My compliments to the authors for the interesting and rare case report. I approve it for pubblication in F1000 Research.

 Please give more explanation for the 10 days delay between patient admission and operative surgical management. On the basis of rare case report the authors should clarify the operative technique with more details.

I have read this submission. I believe that I have an appropriate level of expertise to confirm that it is of an acceptable scientific standard.

# Peer review: Referee response for version 1

**DOI:** 10.5256/f1000research.13246.r27856

**Published:** 2017-11-20

**Authors:** Matteo Novello, Salomone Di Saverio

**Affiliations:** 1 Colorectal Surgery Department, Cleveland Clinic, Cleveland, OH, USA; 2 General Surgery Department, Ospedale Maggiore, Bologna, Italy

The authors should clarify the reasons for the 10 days delay in the operative management of their patient.

 More insight should be provided on the choice of aggressive operative treatment as compared to a percutaneous drainage. Perhaps the drainage would have been more readily available and also less expensive as compared to elective surgery. Keeping in mind the age of the patient, it might have been a reasonable option proceeding with conservative management and percutaneous drainage trial.

 Did the authors investigate the use of somatostatin (provided in the form of Octreotide) in such case? It is currently a first line approach for uncomplicated pancreatic-cutaneous fistulas and might as well work with the Gallbladder considering the physiological functions of the hormone.

 All in all the case is interesting and, considering the rarity of the event, it deserves publication.

We have read this submission. We believe that we have an appropriate level of expertise to confirm that it is of an acceptable scientific standard.
